# Effectiveness of the sleep apnea-specific hypoxic burden and sleep breathing impairment index in assessing cognitive impairment in children with obstructive sleep apnea

**DOI:** 10.3389/fped.2025.1628961

**Published:** 2025-07-24

**Authors:** Simin Zhu, Yanuo Zhou, Chendi Lu, Zitong Wang, Lina Ma, Xiaoxin Niu, Yushan Xie, Zihan Xia, Yonglong Su, Yuqi Yuan, Jiayi Yang, Rui Lu, Xinru Lv, Wei Hou, Yani Feng, Xiaoyong Ren, Yewen Shi

**Affiliations:** Department of Otorhinolaryngology-Head and Neck Surgery, The Second Affiliated Hospital of Xi'an Jiaotong University, Xi’an, China

**Keywords:** sleep apnea-specific hypoxic burden, sleep breathing impairment index, children OSA, cognition impairment, obstructive apnea-hypopnea index

## Abstract

**Purpose:**

This study investigated cognitive impairment in children with obstructive sleep apnea (OSA) by evaluating the utility of sleep apnea-specific hypoxic burden (SASHB) and sleep breathing impairment index (SBII) compared to the obstructive apnea-hypopnea index (OAHI).

**Methods:**

A retrospective analysis included 141 children with suspected OSA from Xi'an Jiaotong University Second Affiliated Hospital (October 2021–October 2024), categorized into OSA (*n* = 104) and non-OSA (*n* = 37) groups based on OAHI. Demographic, polysomnography (PSG), and event-related potential (ERP) data were collected. Cognitive function (full, verbal, and performance IQ: FIQ, VIQ, PIQ) was assessed using the China-Wechsler Intelligence Scale (C-WISC). Correlations between cognitive scores, ERP parameters, OAHI, SASHB, and SBII were analyzed.

**Results:**

OSA children exhibited higher rates of snoring/sleep suffocation, prolonged apnea/hypoventilation durations, reduced mean/minimum SaO2, lower REM sleep, and elevated N3 sleep. OAHI and SASHB were higher in the OSA group, but SBII showed no group difference. OSA children had prolonged P300/N100 latencies and lower FIQ, VIQ, and PIQ scores. FIQ inversely correlated with OAHI, SASHB, and SBII; after adjusting for age, sex, and BMI, FIQ remained negatively associated with SBII. SASHB correlated with FIQ only in children <6 years, while OAHI showed no significant correlation. VIQ in younger children negatively correlated with SASHB/SBII, but only with OAHI in older children. PIQ in younger children correlated with OAHI, while no correlations existed in older children. P300 latency positively correlated with OAHI; other ERP parameters showed no associations.

**Conclusion:**

OSA children demonstrate significant cognitive decline and ERP abnormalities. SASHB and SBII exhibit stronger correlations with cognitive impairment than OAHI, particularly in younger children, highlighting their potential as precise metrics for evaluating cognitive deficits in pediatric OSA.

## 1 Introduction

Pediatric obstructive sleep apnea (OSA) is a common sleep disorder, with an incidence of approximately 1%–6% (1). OSA in children is closely related to cognitive impairment, with research showing that children with OSA typically performing poorly on neurocognitive assessments, including those of executive function, memory, attention, and language expression and comprehension (2). Moreover, cognitive impairment tends to worsen as the severity of OSA increases (3). Prospective studies have indicated that children with OSA show significant improvements in language and motor fluency, sustained attention, and vocabulary 1 year after undergoing adenoid and tonsillectomy (4).

The specific mechanisms underlying cognitive dysfunction due to pediatric OSA remain unclear, but intermittent hypoxia and sleep fragmentation are generally considered related to cognitive impairment. Research has shown that children who experience hypoxemia are more likely to develop cognitive dysfunction compared with those who do not, and the degree of impairment is significantly correlated with the child's daytime oxygen pressure and nighttime blood oxygen saturation (5). Additionally, disruptions in nighttime sleep structure are considered a major cause of cognitive dysfunction in children with OSA (6). However, cognitive impairments related to pediatric OSA are often subtle and can be easily overlooked in clinical diagnosis and treatment.

Currently, the obstructive apnea-hypopnea index (OAHI) is a commonly used clinical measure for diagnosing and assessing the severity of pediatric OSA. However, the OAHI only describes the frequency of breathing events during sleep and does not account for the degree of hypoxemia associated with these events, limiting its ability to reflect the true characteristics and pathological mechanisms of the disease. The sleep apnea-specific hypoxic burden (SASHB) and sleep breathing impairment index (SBII) are newly derived measurement indicators from polysomnography (PSG) results. In contrast to the OAHI, these indexes integrate the characteristics of hypoxemia related to OSA breathing events, allowing for a better understanding of the characteristic pathological mechanisms of OSA. Both indexes have shown potential in identifying high-risk populations for OSA and can be used to predict the risk of cardiovascular events (7–9). However, no research, to our knowledge, has examined the relationship among the SASHB, SBII, and cognitive function in children.

This study aimed to explore cognitive changes in children with obstructive sleep apnea (OSA) from multiple perspectives by analyzing the effectiveness of the sleep apnea-specific hypoxic burden (SASHB) and sleep breathing impairment index (SBII) in assessing cognitive impairment in children with OSA.

The aim of this study was to fill this research gap by exploring cognitive impairment in children with OSA from multiple perspectives, analyzing the effectiveness of the SASHB and SBII in assessing cognitive damage in children. Additionally, the advantages of the SASHB and SBII in evaluating cognitive damage in children was investigated by comparing their use with that of the OAHI.

## 2 Materials and methods

### 2.1 Patients and materials

This study was a retrospective analysis of 141 children who had visited the Department of Otorhinolaryngology-Head and Neck Surgery at the Second Affiliated Hospital of Xi'an Jiaotong University from October 2021 to October 2024 with suspected OSA. OAHI was used to assess the severity of sleep-disordered breathing, which is defined as the number of obstructive apneas, hypopneas, and mixed apneas per hour during sleep. Demographic characteristics, clinical symptoms, signs, event-related evoked potentials (ERPs), and China-Wechsler Intelligence Scale (C-WISC) scores were obtained from medical records, and written informed consent was provided by the children's legal guardians. The inclusion criteria were (1) aged 3–12 years; (2) suspected OSA; and (3) underwent PSG, ERP, and C-WISC testing. The exclusion criteria were (1) missing baseline data; (2) craniofacial disease; (3) receipt of OSA treatment, including medication, surgery, or continuous positive airway pressure (CPAP) therapy; and (4) comorbidities such as Down syndrome, Crouzon syndrome, or Pierre Robin sequence. OAHI was used to assess the severity of sleep-disordered breathing. Participants underwent polysomnography (PSG) for OAHI-derived OSA severity grading. Diagnostic classification followed: (1) Normal: OAHI <1/hr, (2) OSA: OAHI ≥1/hr.

### 2.2 Polysomnography evaluation

All children underwent PSG testing (Computer Medics E-series from Computer Medics Inc., Abbotsford, Australia) that was performed in the hospital. The children and their parents were placed in a dark, quiet room, where the children fell asleep naturally between 22:00 and 23:00 without pharmacological induction and were awakened between 6:00 and 7:00 (10). The PSG was evaluated by two sleep experts, who measured the following parameters: sleep efficiency, average apnea duration, longest apnea duration, average hypoventilation duration, longest hypoventilation duration, mean SaO2, minimum SaO2, REM, Stage N1, Stage N2, Stage N3, and OAHI.

### 2.3 C-WISC and ERP evaluation

Cognitive function was evaluated using the C-WISC, which is based on the WISC and has been revised by Chinese scholars to assess the intelligence of Chinese children (10). For children younger than 6 years, the version of the C-WISC for younger children was used; for those 6 years and older, the children's version of the C-WISC was used (11). All assessments were conducted by clinical psychologists.

ERP data were acquired from participants seated in an electromagnetically shielded room under eyes-closed, relaxed conditions. Using a NeuroCare NTS-2000 system with International 10–20 electrode placement, signals were recorded at Cz (needle electrodes: austenitic stainless steel) referenced to right mastoid. Impedances were kept <5 kΩ. Auditory stimuli (85 dB SPL, 10–30 Hz bandpass) followed an oddball sequence (10% targets). Component windows: N100 (75–150 ms; negative peak), P300 (250–500 ms; positive peak). Peak latency and amplitude were measured for both components.

### 2.4 SASHB and SBII calculation

The SASHB and SBII were calculated based on algorithms from Dr. Azarbarzin's team (12) and Dr. Xiao's team (8) using raw PSG data for measurement. SASHB is defined as the total area under the baseline SpO_2_ curve corresponding to respiratory events per hour during sleep. SBII is defined as the sum of the product of the duration of breathing events and the corresponding desaturation area per hour during sleep. SASHB and SBII was calculated by a customized and automated program managed by python.

### 2.5 Statistical analysis

Data on the clinical characteristics of the participants were processed and analyzed using R software (version 4.3.2) and SPSS 26.0 (IBM Corporation, Armonk, NY, USA). Categorical variables were expressed as numbers (n) and percentages (%), and between-group comparisons were performed using the chi-square test and Fisher's exact test. The normality of continuous variables was tested using the Shapiro–Wilk test. Data that followed a normal distribution were analyzed using a *t* test and expressed as the mean ± standard deviation. Non-normally distributed data were analyzed using non-parametric tests, and the results were presented as the median and interquartile range. After data cleaning and feature variable selection from the clinical data, *p* ≤ *0.05 was* considered statistically significant.

## 3 Results

### 3.1 Comparison of demographic characteristics, symptoms, signs, and PSG parameters of the OSA and non-OSA groups

After strictly applying the inclusion and exclusion criteria, a total of 141 participants, 104 children with OSA and 37 children without OSA, were included in this study (Table 1). Among them, boys accounted for 70.21% (99) and girls for 29.79% (27), with no significant gender difference between the two groups (*p* = 0.669). The proportion of children who experience snoring and sleep suffocation in the OSA group, as reported by their guardians, was significantly higher than that of children who experience these symptoms in the non-OSA group (both *p* < 0.05). There were no significant differences between the two groups in terms of age, body mass index (BMI), mouth breathing, or Epworth Sleepiness Scale scores.

**Table 1:** Comparison of demographic characteristics, symptoms, signs, and PSG parameters of the OSA and non-OSA groups.

Variables	Total (*n* = 141)	Normal (*n* = 37)	OSA (*n* = 104)	*p*
Sex, n(%)				0.669
Girl	42 (29.79)	10 (27.03)	32 (30.77)	
Boy	99 (70.21)	27 (72.97)	72 (69.23)	
Age, years, M (Q₁, Q₃)	6.00 (5.00, 8.00)	7.00 (5.00, 9.00)	6.00 (5.00, 8.00)	0.866
BMI, kg/m², M (Q₁, Q₃)	15.70 (14.70, 17.80)	15.10 (14.40, 17.50)	15.70 (14.70, 17.88)	0.202
Snoring, *n*(%)				**
No	14 (9.93)	9 (24.32)	5 (4.81)	
Yes	127 (90.07)	28 (75.68)	99 (95.19)	
Mouth breathing, *n*(%)				0.396
No	19 (13.48)	7 (18.92)	12 (11.54)	
Yes	122 (86.52)	30 (81.08)	92 (88.46)	
Sleep suffocating, *n*(%)				*
No	66 (46.81)	23 (62.16)	43 (41.35)	
Yes	75 (53.19)	14 (37.84)	61 (58.65)	
Epworth, M (Q₁, Q₃)	3.00 (1.00, 6.00)	3.00 (1.00, 4.00)	4.00 (2.00, 6.00)	0.092
Sleep efficiency, %, M (Q₁, Q₃)	93.50 (87.30, 96.10)	95.20 (88.90, 97.00)	92.95 (86.47, 95.75)	0.073
Average apnea duration, s, M (Q₁, Q₃)	10.70 (9.00, 12.20)	10.10 (8.80, 11.70)	10.75 (9.30, 12.40)	0.158
Longest apnea duration, s, M (Q₁, Q₃)	16.50 (13.00, 22.00)	14.50 (12.00, 18.00)	17.75 (13.50, 23.62)	**
Average hypoventilation duration, s, M (Q₁, Q₃)	12.40 (8.80, 15.60)	0.00 (0.00, 12.00)	13.20 (10.30, 16.50)	***
Longest hypoventilation duration, s, M (Q₁, Q₃)	17.00 (10.50, 25.50)	0.00 (0.00, 13.00)	19.50 (13.50, 33.50)	***
Mean SaO2, %, M (Q₁, Q₃)	97.00 (97.00, 98.00)	97.00 (97.00, 98.00)	97.00 (96.00, 98.00)	*
Minimum SaO2, %, M (Q₁, Q₃)	88.00 (85.00, 90.00)	90.00 (88.00, 92.00)	87.00 (84.75, 90.00)	***
REM, %, M (Q₁, Q₃)	18.70 (13.80, 22.50)	20.00 (15.50, 23.90)	17.05 (13.40, 21.93)	*
Stage N1, %, M (Q₁, Q₃)	4.60 (2.60, 7.00)	4.00 (2.30, 5.80)	4.75 (2.70, 7.12)	0.308
Stage N2, %, Mean ± SD	48.30 ± 10.26	49.59 ± 10.33	47.85 ± 10.25	0.377
Stage N3, %, Mean ± SD	26.62 ± 9.34	22.58 ± 7.92	28.06 ± 9.41	**
OAHI, M (Q₁, Q₃)	2.40 (0.99, 5.37)	0.40 (0.20, 0.60)	3.10 (1.97, 7.95)	***
SASHB, M (Q₁, Q₃)	0.95 (0.23, 1.80)	0.24 (0.06, 0.63)	1.19 (0.44, 2.43)	***
SBII, M (Q₁, Q₃)	0.32 (0.07, 0.74)	0.24 (0.04, 0.63)	0.35 (0.09, 1.03)	0.223

OSA, obstructive sleep apnea; BMI, body mass index; OAHI, obstructive apnea-hypopnea index; SASHB, sleep apnea-specific hypoxic burden; SBII, sleep breathing impairment index; M, median; Q₁, 1st Quartile; Q₃, 3st Quartile.

* *p* < 0.05.

** *p* < 0.01.

*** *p* < 0.001.

Regarding PSG parameters (Table 1), the OSA group experienced a longer longest apnea duration (14.50 vs. 17.75), a longer average hypoventilation duration (0.00 vs. 13.20), and a longer longest hypoventilation duration (0.00 vs. 19.50) (all *p* < 0.05). The mean SaO_2_ (*p* < 0.05) and minimum SaO_2_ (*p* < 0.001) were lower in the OSA group. In terms of sleep stages, the proportion of REM sleep was lower (20.00 vs. 17.05, *p* < 0.05) while the proportion of Stage N3 sleep was higher (22.58 vs. 28.06, *p* < 0.01) in the OSA group. Although the OSA group had a larger OAHI value (0.40 vs. 3.10, *p* < 0.001) and SASHB value (0.24 vs. 1.19, *p* < 0.001), there was no significant difference in SBII values between the two groups (*p* = 0.223).

### 3.2 Comparison of C-WISC and ERP parameters between OSA and non-OSA groups

Compared with the non-OSA group, the OSA group had a higher P300 amplitude (11.67 vs. 13.09, *p* < 0.05) and N100 amplitude (5.99 vs. 9.28, *p* < 0.01) (Table 2). In contrast, there were no significant differences in P300 latency or N100 latency between the OSA and non-OSA groups. Regardless of whether the children were older or younger than 6 years, the full intelligence quotient (FIQ) and verbal IQ (VIQ) of the OSA group were significantly lower (both *p* < 0.05). There was no significant difference in performance IQ (PIQ) between the two groups in children younger than 6 years, whereas the PIQ of the OSA group was lower in children older than 6 years (*p* < 0.05).

**Table 2:** Comparison of C-WISC and ERP parameters between OSA and non-OSA groups.

Variables	Total (*n* = 141)	Normal (*n* = 37)	OSA (*n* = 104)	*p*
P300 latency, ms, M (Q₁, Q₃)	327.50 (300.00, 355.17)	312.07 (283.30, 345.97)	328.45 (300.00, 357.83)	0.124
P300 amplitude, uV, M (Q₁, Q₃)	12.50 (9.30, 17.68)	11.67 (7.94, 15.02)	13.09 (9.62, 17.87)	*
N100 latency, ms, M (Q₁, Q₃)	108.35 (100.00, 117.24)	106.89 (100.00, 113.79)	110.00 (100.00, 118.32)	0.267
N100 amplitude, uV, M (Q₁, Q₃)	8.75 (4.49, 13.51)	5.99 (4.01, 9.41)	9.28 (5.13, 14.12)	**
FIQ, M (Q₁, Q₃)				
Younger than 6 years	99.00 (92.75, 103.00)	102.50 (99.25, 108.75)	98.50 (90.00, 101.00)	**
Older than 6 years	106.00 (100.00, 112.00)	111.00 (102.50, 116.00)	105.00 (97.25, 109.75)	*
VIQ, M (Q₁, Q₃)				
Younger than 6 years	101.00 (91.00, 107.00)	105.00 (97.25, 111.00)	101.00 (89.00, 107.00)	*
Older than 6 years	105.00 (95.00, 109.00)	111.00 (103.00, 116.00)	101.00 (93.00, 106.00)	**
PIQ, M (Q₁, Q₃)				
Younger than 6 years	91.50 (84.75, 98.00)	93.50 (91.00, 98.75)	90.00 (82.25, 98.00)	0.056
Older than 6 years	98.00 (90.00, 108.00)	106.00 (99.00, 113.00)	98.00 (89.25, 106.75)	*

OSA, obstructive sleep apnea; BMI, body mass index; OAHI, obstructive apnea-hypopnea index; SASHB, sleep apnea-specific hypoxic burden; SBII, sleep breathing impairment index; FIQ, full-scale IQ; PIQ, performance IQ; VIQ, verbal IQ; M, median; Q₁, 1st Quartile; Q₃, 3st Quartile.

* *p* < 0.05.

** *p* < 0.01.

### 3.3 Comparison of PSG parameters among children with different cognition levels

The participants were divided into four groups based on the interquartile range of cognition. Tables 3, 4 show the comparison of PSG parameters among the different cognition groups for children younger and older than 6 years, respectively. Among the children younger than 6 years, as the FIQ increased, both the OAHI and SBII showed a decreasing trend (*p* < 0.05). However, neither OAHI nor SBII demonstrated significant decreasing trends between Q1–Q2 and Q2–Q3 subgroups, although there was no significant trend in the SASHB. In children older than 6 years, the OAHI, SASHB, and SBII all showed a decreasing trend (all *p* < 0.05).

**Table 3:** Comparison of PSG parameters among children younger than 6 years with different cognition levels.

Variables	Total	FIQ ≤92.75	92.75 < FIQ ≤97.28	97.28 < FIQ ≤103.00	103.00< FIQ	*p*
		(FIQ ≤ Q₁)	(Q_1_ < FIQ ≤ Q_2_)	(Q_2_ < FIQ ≤ Q_3_)	(Q_3_ < FIQ)	
	(*n* = 76)	(*n* = 19)	(*n* = 14)	(*n* = 23)	(*n* = 20)	
Sleep efficiency, %, M (Q₁, Q₃)	94.65 (87.90, 96.75)	95.60 (92.50,97.85)	94.65 (87.15,96.80)	91.60 (84.50,95.65)	95.60 (94.30,97.12)	0.137
Average apnea duration, s, M (Q₁, Q₃)	10.95 (9.38, 12.22)	11.00 (10.25,12.20)	11.45 (8.83,12.25)	11.90 (10.25,12.75)	10.10 (8.82,11.50)	0.167
Longest apnea duration, s, M (Q₁, Q₃)	17.75 (14.00, 22.75)	17.50 (15.25,22.00)	16.75 (12.12,23.12)	20.50 (15.25,25.75)	15.50 (13.38,18.88)	0.286
Average hypoventilation duration, s, M (Q₁, Q₃)	12.35 (8.88, 15.60)	13.20 (10.20,15.80)	11.40 (2.45,13.92)	13.30 (9.50,17.45)	11.55 (0.00,12.53)	0.245
Longest hypoventilation duration, s, M (Q₁, Q₃)	17.00 (10.38, 25.62)	20.50 (13.25,32.00)	15.50 (2.62,22.88)	22.50 (12.75,33.25)	12.50 (0.00,17.88)	0.120
Mean SaO2, %, M (Q₁, Q₃)	97.00 (96.00, 98.00)	97.00 (96.00,97.00)	97.00 (96.25,98.00)	97.00 (96.00,98.00)	97.00 (97.00,98.00)	0.541
Minimum SaO2, %, M (Q₁, Q₃)	87.50 (84.75, 90.00)	86.00 (81.50,88.00)	88.00 (86.00,89.75)	87.00 (82.50,90.00)	88.00 (86.75,90.00)	0.212
REM, %, M (Q₁, Q₃)	18.20 (13.47, 22.50)	19.20 (13.45,22.05)	18.15 (15.85,23.27)	15.30 (11.90,23.25)	15.95 (13.78,20.67)	0.853
Stage N1, %, M (Q₁, Q₃)	4.15 (1.80, 6.60)	4.00 (1.80,5.65)	1.95 (1.45,7.17)	5.00 (3.80,7.20)	4.00 (1.75,6.45)	0.231
Stage N2, %, Mean ± SD	49.30 (40.70, 54.68)	51.00 (41.85,61.35)	52.15 (40.90,56.85)	48.90 (37.05,51.90)	47.15 (42.08,53.02)	0.561
Stage N3, %, Mean ± SD	26.70 (20.45, 32.50)	27.80 (22.55,31.10)	24.40 (19.33,32.32)	27.40 (21.70,32.40)	28.10 (20.50,33.80)	0.956
OAHI, M (Q₁, Q₃)	2.50 (1.23, 7.27)	3.31 (2.13,9.80)	2.14 (0.93,6.67)	2.96 (1.71,11.36)	1.46 (0.40,2.50)	*
SASHB, M (Q₁, Q₃)	1.20 (0.47, 3.46)	1.83 (1.18,4.60)	0.91 (0.23,2.80)	1.35 (0.32,4.74)	1.05 (0.47,1.49)	0.067
SBII, M (Q₁, Q₃)	0.35 (0.12, 0.66)	1.12 (0.39,2.40)	0.34 (0.08,0.55)	0.36 (0.18,0.62)	0.13 (0.03,0.28)	***

OAHI, obstructive apnea-hypopnea index; SASHB, sleep apnea-specific hypoxic burden; SBII, sleep breathing impairment index; C-WISC, China-Wechsler intelligence scale, FIQ, full-scale IQ; M, median; Q₁, 1st Quartile; Q_2_, 2st Quartile; Q₃, 3st Quartile.

* *p* < 0.05.

** *p* < 0.01.

*** *p* < 0.001.

**Table 4:** Comparison of PSG parameters among children older than 6 years with different cognition levels.

Variables	Total	FIQ ≤95.00	95.00 < FIQ ≤ 101.00	101.00 < FIQ ≤ 109.00	109.00 < FIQ	*p*
		(FIQ ≤ Q₁)	(Q_1_ < FIQ ≤ Q_2_)	(Q_2_ < FIQ ≤ Q_3_)	(Q_3_ < FIQ)	
	(*n* = 65)	(*n* = 15)	(*n* = 17)	(*n* = 16)	(*n* = 17)	
Sleep efficiency, %, M (Q₁, Q₃)	91.30 (86.50, 95.20)	92.45 (89.82,93.93)	90.00 (86.50,96.25)	91.35 (86.20,95.25)	92.10 (84.82,95.03)	0.996
Average apnea duration, s, M (Q₁, Q₃)	10.30 (8.80, 11.60)	9.70 (9.00,10.55)	10.90 (9.55,11.90)	9.40 (8.10,12.45)	10.75 (9.27,11.40)	0.676
Longest apnea duration, s, M (Q₁, Q₃)	16.00 (12.00, 20.50)	16.75 (11.50,20.12)	16.50 (12.25,22.50)	15.25 (12.38,23.25)	15.75 (13.12,18.38)	0.956
Average hypoventilation duration, s, M (Q₁, Q₃)	12.40 (8.80, 15.80)	12.35 (9.80,14.90)	12.70 (4.40,14.55)	12.85 (10.15,17.42)	12.50 (8.03,15.83)	0.817
Longest hypoventilation duration, s, M (Q₁, Q₃)	17.00 (11.50, 21.50)	18.00 (13.62,20.62)	19.00 (6.50,22.50)	18.50 (11.50,34.00)	13.25 (9.75,18.50)	0.383
Mean SaO2, %, M (Q₁, Q₃)	97.00 (97.00, 98.00)	97.00 (97.00,97.25)	97.00 (96.00,98.00)	97.00 (97.00,97.25)	97.00 (97.00,98.00)	0.438
Minimum SaO2, %, M (Q₁, Q₃)	89.00 (86.00, 91.00)	88.50 (85.75,90.00)	89.00 (83.50,92.00)	88.00 (84.25,90.25)	90.50 (89.25,92.00)	0.053
REM, %, M (Q₁, Q₃)	19.00 (14.60, 22.40)	20.10 (16.50,22.03)	19.20 (12.15,21.10)	18.25 (15.13,21.50)	17.50 (13.85,24.00)	0.739
Stage N1, %, M (Q₁, Q₃)	4.90 (3.20, 7.00)	4.65 (3.05,5.78)	5.20 (3.75,7.55)	4.95 (3.77,7.95)	5.50 (3.75,6.57)	0.584
Stage N2, %, Mean ± SD	46.80 (42.80, 55.50)	46.80 (44.03,56.33)	45.90 (42.45,49.40)	48.45 (39.52,57.45)	48.85 (45.95,55.00)	0.592
Stage N3, %, Mean ± SD	25.40 (19.30, 32.20)	23.45 (18.20,29.77)	25.40 (21.90,35.05)	25.85 (19.12,32.30)	28.25 (18.82,31.08)	0.725
OAHI, M (Q₁, Q₃)	2.00 (0.82, 3.80)	3.03 (1.53,4.85)	2.50 (1.01,3.42)	2.29 (1.20,5.88)	0.96 (0.43,1.54)	*
SASHB, M (Q₁, Q₃)	0.35 (0.07, 1.19)	0.89 (0.28,1.17)	0.74 (0.11,3.12)	0.45 (0.08,1.23)	0.08 (0.04,0.31)	*
SBII, M (Q₁, Q₃)	0.29 (0.04, 0.94)	0.79 (0.26,2.41)	0.58 (0.08,1.11)	0.12 (0.03,0.40)	0.05 (0.01,0.25)	**

OAHI, obstructive apnea-hypopnea index; SASHB, sleep apnea-specific hypoxic burden; SBII, sleep breathing impairment index; C-WISC, China-Wechsler intelligence scale, FIQ, full-scale IQ; M, median; Q₁, 1st Quartile; Q_2_, 2st Quartile; Q₃, 3st Quartile.

* *p* < 0.05.

** *p* < 0.01.

### 3.4 C-WISC and ERP parameter correlation analysis

**Figure 1:**
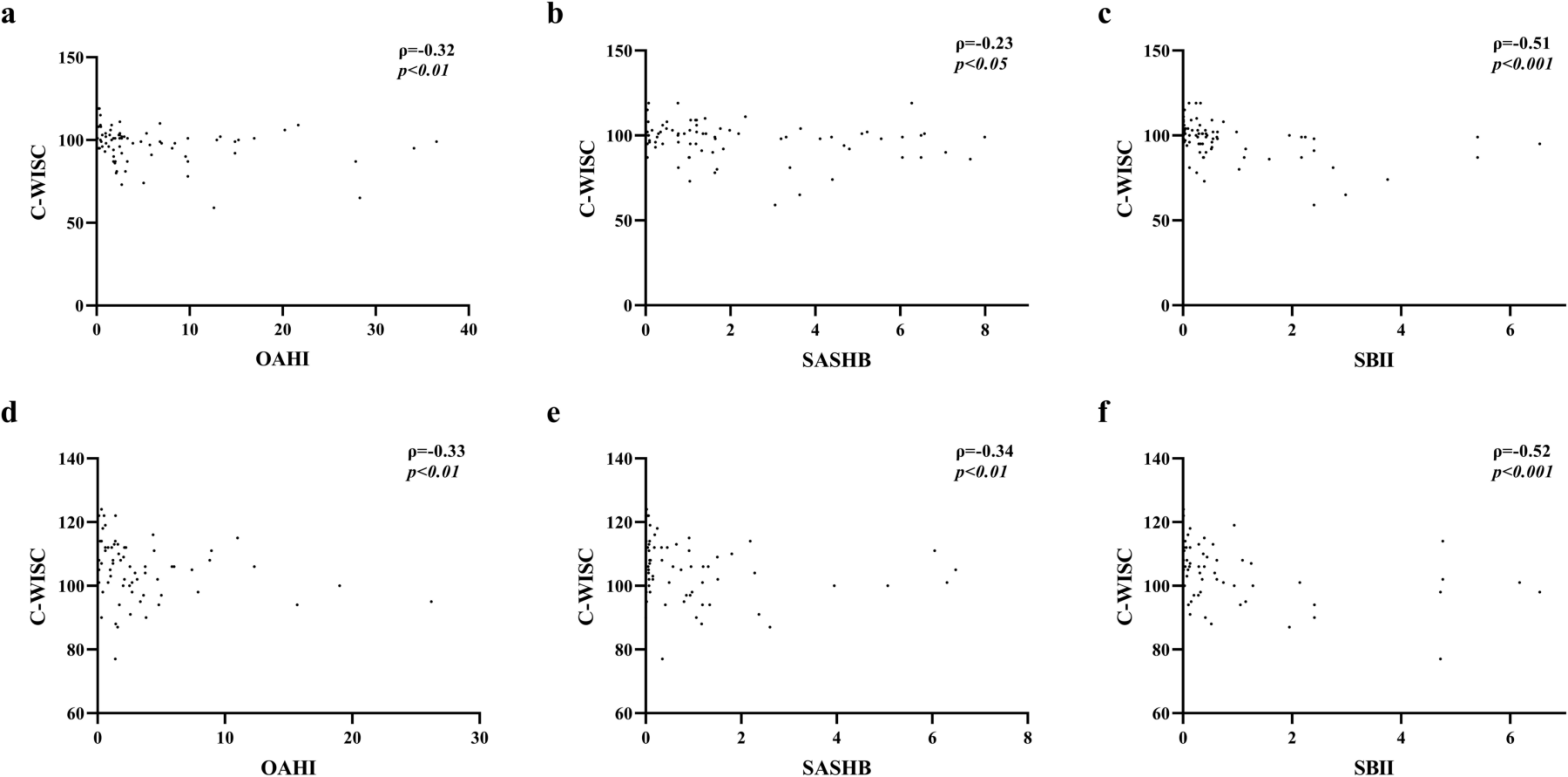
Correlation between FIQ and OAHI, SASHB, SBII. **(a–c)** Correlation between FIQ scores and OAHI, SASHB, and SBII values in children younger than 6 years. **(d–f)** Correlation between FIQ scores and OAHI, SASHB, and SBII values in children older than 6 year.

Correlation analysis of the OAHI, SASHB, SBII, and FIQ (Figure 1) revealed that in both children older and younger than 6 years, the FIQ was negatively correlated with the OAHI, SASHB, and SBII. After adjusting for age, sex, and BMI, the FIQ remained negatively correlated with the SBII (*p* < 0.01), while the SASHB was negatively correlated with the FIQ only in children younger than 6 years (*r* = −0.22, *p* < 0.01). Although no significant correlation was found between the OAHI and FIQ, the VIQ was negatively correlated with the OAHI, SASHB, and SBII. After adjusting for age, sex, and BMI, in children younger than 6 years, the VIQ remained negatively correlated with the SASHB (*r* = −0.29, *p* < 0.05) and SBII (*r* = −0.29, *p* < 0.05). However, in children older than 6 years, the VIQ was negatively correlated only with the OAHI (*r* = −0.27, *p* < 0.05). The PIQ was negatively correlated with the OAHI (*r* = −0.31, *p* < 0.01) and SBII (*r* = −0.26, *p* < 0.05) in children younger than 6 years but showed no correlation with the OAHI, SASHB, or SBII in children older than 6 years. Although P300 latency was positively correlated with the OAHI (*r* = 0.17, *p* < 0.05), P300 amplitude, N100 latency, and N100 amplitude showed no significant correlation with the OAHI, SASHB, or SBII (Table 5).

**Table 5:** Correlation analysis of OAHI, SASHB, SBII with C-WISC and ERP parameters.

	OAHI				SASHB				SBII			
	*ρ*	*p*	r	*p*	ρ	*p*	r	*p*	ρ	*p*	r	*p*
P300 latency	0.16	0.052	0.17	*	0.18	*	0.09	0.294	0.10	0.239	−0.02	0.819
P300 amplitude	0.13	0.120	0.06	0.511	0.13	0.140	0.05	0.539	0.02	0.788	0.08	0.348
N100 latency	0.06	0.502	0.06	0.463	0.16	0.061	0.17	0.052	0.03	0.720	0.06	0.469
N100 amplitude	0.17	*	0.02	0.804	0.07	0.417	0.02	0.800	0.04	0.635	0.04	0.626
FIQ												
Younger than 6 years	−0.32	**	−0.23	0.051	−0.23	*	−0.22	**	−0.51	***	−0.40	***
Older than 6 years	−0.33	**	−0.21	0.102	−0.34	**	−0.17	0.187	−0.52	***	−0.39	**
VIQ												
Younger than 6 years	−0.16	0.182	−0.14	0.254	−0.29	**	−0.29	*	−0.26	*	−0.29	*
Older than 6 years	−0.38	**	−0.27	*	−0.26	*	−0.13	0.309	−0.37	**	−0.16	0.217
PIQ												
Younger than 6 years	−0.33	**	−0.31	**	−0.11	0.362	−0.16	0.175	−0.31	**	−0.26	*
Older than 6 years	−0.23	0.072	−0.14	0.292	−0.20	0.110	−0.06	0.655	−0.06	0.621	0.09	0.505

OAHI, obstructive apnea-hypopnea index; SASHB, sleep apnea-specific hypoxic burden; SBII, sleep breathing impairment index; C-WISC, China-Wechsler intelligence scale, FIQ, full-scale IQ, PIQ, performance IQ; VIQ, verbal IQ.

* *p* < 0.05.

** *p* < 0.01.

*** *p* < 0.001.

## 4 Discussion

Cognitive impairment related to pediatric OSA is often overlooked in clinical diagnosis and treatment, highlighting the need to thoroughly investigate cognitive deficits in children with OSA. This study of the effectiveness of the SASHB and SBII in assessing cognitive dysfunction in children with OSA was the first to explore the differences in SASHB and SBII values between children with and without OSA.

Unlike the OAHI, the traditional indicator of OSA, the SASHB and SBII incorporate blood oxygen data from PSG (7, 13), thereby providing a better reflection of the characteristic pathological and physiological changes during the occurrence of OSA. In our examination of these indexes, we observed for the first time that the SASHB and SBII are correlated with cognitive function in children with OSA and are superior to the OAHI in assessing OSA.

In our study, the proportion of children experiencing snoring and sleep suffocation as reported by their guardians was higher in children with OSA than in children without OSA, which is consistent with previous studies (14). However, unlike prior research, we found no significant differences in BMI or mouth breathing between children with and without OSA. We speculate that this may be due to the relatively small sample size in this study (15). Regarding PSG parameters, children with OSA experienced longer longest apnea duration, longer average hypoventilation duration, and longer longest hypoventilation duration compared with children without OSA, suggesting that respiratory pauses during sleep might be more severe in children with OSA (16). Children with OSA also had lower mean SaO_2_ and minimum SaO_2_, which aligns with previous findings (17), indicating that oxygen levels are lower during sleep in children with OSA. Interestingly, the proportion of REM sleep was lower and Stage N3 was higher in children with OSA, while there were no significant differences in Stage N1 and Stage N2 sleep between children with and without OSA. Li et al. found that compared with preschool children, school-age children experience more pronounced changes in sleep structure (18), with higher percentages of Stage N1 and Stage N2 sleep and lower percentages of Stage N3 and REM sleep. This suggests that sleep structure changes may play a significant role in pediatric OSA, which we plan to explore in a future study of the role of sleep stages in pediatric OSA that includes a larger sample stratified by age. Additionally, children with OSA had higher OAHI, SASHB, and SBII values, which is consistent with previous studies (19).

Among the various methods of cognitive assessment, use of the C-WISC, a non-invasive approach, is common for evaluating the cognitive function of Chinese children (10). In this study, children with OSA had lower C-WISC scores, further highlighting the cognitive impairment caused by OSA, which may be due to intermittent hypoxia induced by OSA (20). ERPs have also been used to study cognitive brain function (21), with studies reporting that patients with OSA have prolonged latencies and reduced amplitudes (22, 23), but these findings remain controversial. We found that children with OSA had higher P300 and N100 amplitudes compared with children without OSA but no significant differences in P300 and N100 latencies between the two groups, which contrasts with previous studies (24).The N100 and P300 event-related potentials were observed within 90–200 ms and 250–300 ms post-stimulus onset, respectively. The P300 component reflects higher-order cognitive processing that is independent of the physical attributes of sensory input. Our findings demonstrated a significant positive correlation between P300 latency and OAHI, suggesting progressive cognitive impairment in information processing capacity with increasing OSA severity. However, no statistically significant associations were detected between OSA severity and either P300 amplitude, N100 latency, or N100 amplitude. These negative findings may potentially reflect the limited statistical power due to our moderate sample size. Furthermore, the complexity of ERP methodology, including its technical requirements and susceptibility to various confounding factors, may limit its widespread clinical applicability for OSA assessment. Nevertheless, ERPs, as a potential method for detecting OSA-related cognitive impairment, warrant further investigation in the future.

Correlation analysis of the OAHI, SASHB, SBII, and cognitive function revealed that as IQ increased, the OAHI, SASHB, and SBII all showed a decreasing trend, while there were no significant differences in mean SaO2, minimum SaO2, REM, Stage N1, Stage N2, or Stage N3 between those groups. We believe that the OAHI, SASHB, and SBII are comprehensive indicators for evaluating the severity of OSA that provide a more comprehensive reflection of OSA severity compared with individual factors such as blood oxygen levels or sleep stages. After adjusting for age, sex, and BMI, the FIQ remained negatively correlated with the SBII, while the SASHB was negatively correlated with the FIQ only in children younger than 6 years, and the OAHI showed no significant correlation with the FIQ. This suggests that compared with the OAHI, the SASHB and SBII are better indicators of OSA-related cognitive impairment.

Xu et al. found that CPAP treatment improved neurocognitive function in adults with OSA (25), indicating that improvements in blood oxygen levels contribute to cognitive enhancement. Our research also suggests that in children, OSA-related hypoxia may play a key role in cognitive impairment (26, 27). Interestingly, the VIQ in children younger than 6 years was negatively correlated with the SASHB and SBII, but was negatively correlated with the OAHI only in children older than 6 years. The PIQ in children younger than 6 years was negatively correlated with the OAHI and SBII, but showed no correlation with the OAHI, SASHB, or SBII in children older than 6 years. We hypothesize that these findings may be due to older children having higher cognitive levels as their educational attainment increases, leading to the reduced impact of OSA. In future studies, age matching of participants should be performed to further analyze the correlations among the OAHI, SASHB, SBII, and cognitive impairment in children.

## 5 Conclusion

We assessed the cognitive status of children with obstructive sleep apnea through a variety of methods. It was found that children with OSA had lower FIQ, VIQ, and PIQ values and higher P300 and N100 latencies compared with children without OSA. We also explored the correlation between SASHB, SBII, OAHI and children's cognitive level. The SASHB and SBII were more strongly correlated with children's cognitive level than the OAHI. Therefore, we concluded that because these two indicators have important value in the assessment of cognitive impairment in children, it is necessary to place focus on the SASHB and SBII in clinical work to identify children with cognitive impairment at an early stage.

## Data Availability

The raw data supporting the conclusions of this article will be made available by the authors, without undue reservation.
